# Supplementary material for: Stratification of Colorectal Patients Based on Survival Analysis Shows the Value of Consensus Molecular Subtypes and Reveals the CBLL1 Gene as a Biomarker of CMS2 Tumours
Source: Int J Mol Sci. 2024 Feb 5;25(3):1919. doi: 10.3390/ijms25031919 (PMC10856263; doi:10.3390/ijms25031919)
Supplement: Supplementary file 1 [file ijms-25-01919-s001.zip › Supplementary_Figure_S1.pdf]

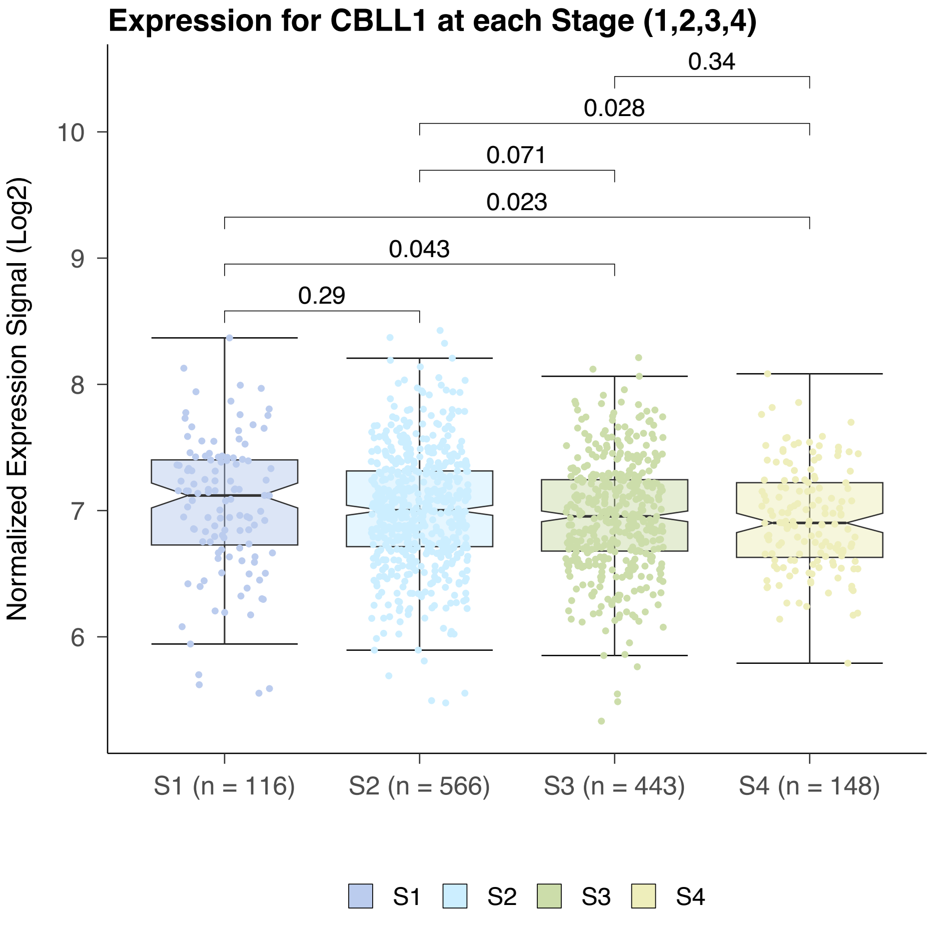

Supplementary Figure S1. Expression of the gene CBLL1 in a cohort of 1273 CRC tumours divided into different stages (from stage 1 to stage 4, S1-S4).
