# Supplementary material for: Stratification of Colorectal Patients Based on Survival Analysis Shows the Value of Consensus Molecular Subtypes and Reveals the CBLL1 Gene as a Biomarker of CMS2 Tumours
Source: Int J Mol Sci. 2024 Feb 5;25(3):1919. doi: 10.3390/ijms25031919 (PMC10856263; doi:10.3390/ijms25031919)
Supplement: Supplementary file 1 [file ijms-25-01919-s001.zip › Supplementary_Figure_S2.pdf]

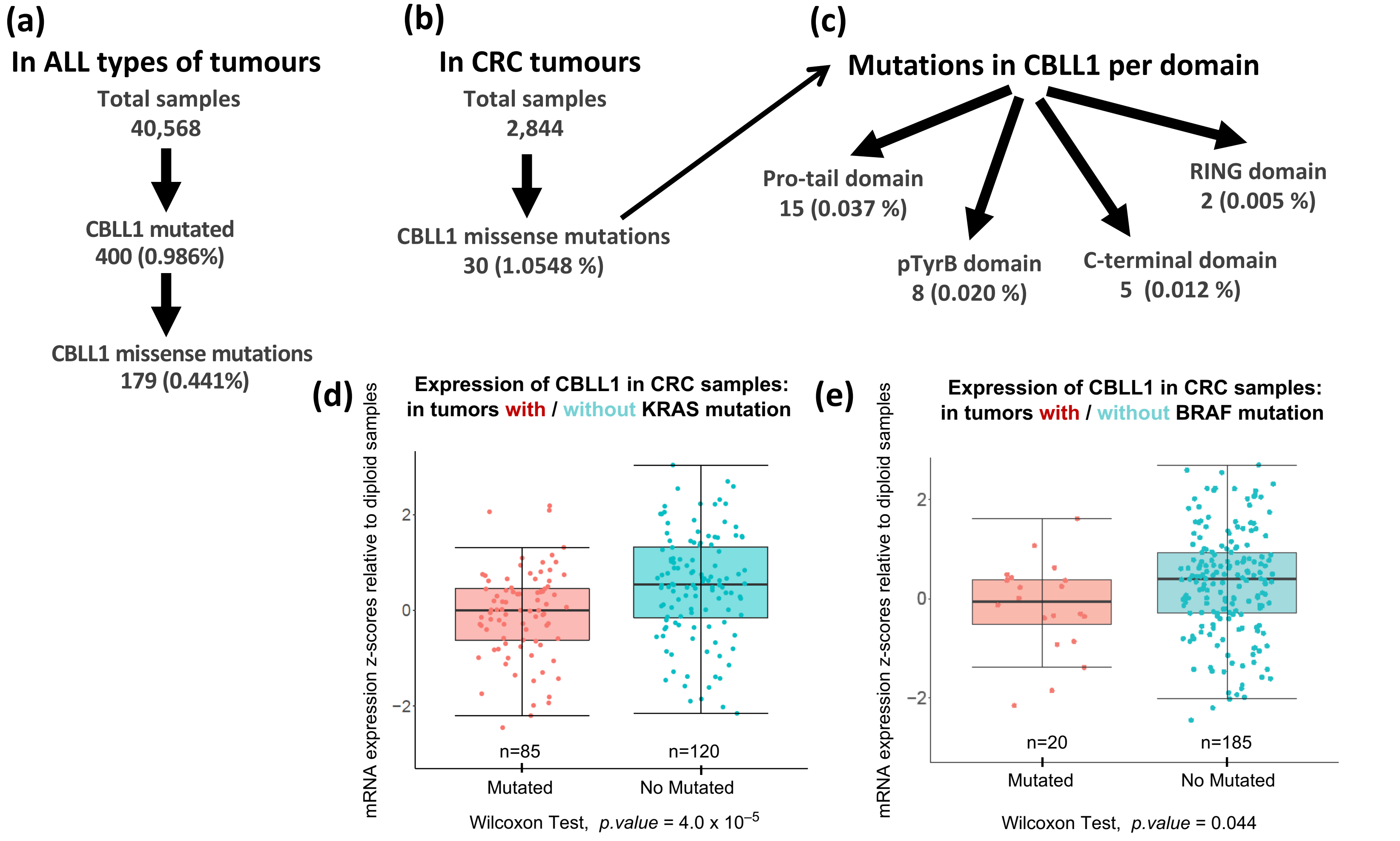

Supplementary Figure S2. Somatic mutations reported for the human gene CBLL1 (taken from COSMIC database, https://cancer.sanger.ac.uk/cosmic, accessed on 10 February 2023): **(a)** in all types of tumours; **(b)** in colorectal cancer tumours; **(c)** in colorectal cancer tumours within the different domains of the protein. Differences in the expression distribution of CBLL1 in CRC samples comparing the tumours with or without mutation **(d)** in KRAS, or **(e)** in BRAF.
